# Increased Urinary IgA in Paediatric IgA Vasculitis Nephritis

**DOI:** 10.3390/ijms232314548

**Published:** 2022-11-22

**Authors:** Julien Marro, Andrew J. Chetwynd, Samuel Edwards, Rachael D. Wright, Louise Oni

**Affiliations:** 1 Department of Women’s and Children’s Health, Institute of Life Course and Medical Sciences, University of Liverpool, Liverpool L12 2AP, UK; 2 Centre for Proteome Research, Institute of Systems, Molecular and Integrative Biology, University of Liverpool, Liverpool L69 7ZB, UK; 3 Department of Paediatric Nephrology, Alder Hey Children’s NHS Foundation Trust Hospital, Liverpool L14 5AB, UK

**Keywords:** IgAV, paediatric, IgAVN, children, kidney, renal

## Abstract

IgA vasculitis (IgAV) is the most common form of paediatric vasculitis, with up to 50% of patients experiencing kidney inflammation. Much remains unknown about IgAV, but it is believed to arise due to galactose-deficient IgA1 promoting an auto-inflammatory response. This study assesses whether urinary IgA can be detected in children with IgAV to allow further evaluation of IgA1 and whether it has any relationship with nephritis. Urinary and serum IgA concentrations were measured using commercially available ELISA kits. Patients were grouped into IgAV nephritis (IgAVN) or IgAV without nephritis (IgAVwoN). Fifty-nine children were included: IgAVN n = 12, IgAVwoN n = 35, and healthy controls (HC) n = 12, with a mean age of 8.2 ± 4.1 years. Urinary IgA concentrations were statistically significantly higher in patients with IgAV (107.1 ± 136.3 μg/mmol) compared to HC (50.6 ± 26.3 μg/mmol; *p* = 0.027) and IgAVN (229.8 ± 226.3 μg/mmol) compared to both IgAVwoN (65.0 ± 37.8 μg/mmol; *p* = 0.002) and HC (*p* < 0.001). Urinary IgA concentrations were able to distinguish between renal status (AUC 0.838, 95%CI [0.704–0.973], *p* < 0.001) and did not correlate with proteinuria (r = 0.124; *p* = 0.407). Urinary IgA concentrations are increased in children with IgAVN, and it has the potential to act as a non-invasive biofluid to further evaluate nephritis in this disease.

## 1. Introduction

IgA vasculitis (IgAV, formerly Henoch-Schönlein purpura, HSP) is the most common form of vasculitis encountered in childhood, with an annual incidence estimated to be around 20 per 100,000 children [1]. It is a multisystemic disease that usually presents as a purpuric, non-blanching rash with lower limb predominance in a previously well child [2]. Other systemic manifestations include abdominal involvement, joint involvement and kidney inflammation that is reported in up to 50% of children [3]. Despite an excellent prognosis in the vast majority of children, nephritis (IgAVN) accounts for the majority of long-term mortality and morbidity, with 1–2% ultimately progressing to kidney failure [4]. Currently, no clinical features or tests are able to identify which patients are at high risk of developing nephritis [2].

The exact pathophysiology of IgAVN remains unknown, although it is likely to result from a combination of environmental factors (i.e., upper respiratory tract infections) and genetic predisposition [2]. IgAV is believed to share a similar pathophysiology with IgA nephropathy (IgAN), and it has strikingly similar histological appearances. Across both IgAVN and IgAN, there is a growing body of evidence that they are driven by abnormalities in the hinge regions of immunoglobulin A1 (IgA1), specifically the O-glycans, which may be aberrantly glycosylated, leading to galactose deficient IgA1 (gd-IgA1) [5,6]. The exposed GalNac residues can then act as neoepitopes and potentially initiate the kidney inflammatory process, starting with mesangial deposition of pathogenic gd-IgA1 [5,7].

As there is a growing interest in gd-IgA1 in IgAVN, the ability to detect, quantify, and extract IgA from a non-invasive biofluid to investigate its relationship with nephritis and its precise glycosylation status is attractive as it may permit earlier stratification of children at high risk of kidney failure. To date, only one previous biomarker study, that evaluated >10 urine and serum cytokines, immunoglobulins, and immune complexes in IgAV has highlighted the potential strength of urinary IgA as a predictor of children with IgAVN (AUC 0.86); however, that study demonstrated a possible correlation with the quantity of protein excreted in the urine (correlation coefficient 0.47) [8]. Further studies are therefore required to confirm these findings and to determine whether this is simply a urinary by-product due to an inflamed glomerulus or whether it may have a more specific relationship with the disease activity. 

The aim of this study was to assess whether urinary IgA could be detected in a cohort of children with IgAV and confirm whether there was any relationship between urinary IgA and the presence of nephritis.

## 2. Results

### 2.1. Paediatric Cohort

A total of 59 children were recruited in this study (IgAVN n = 12, IgAVwoN n = 35, HC n = 12); their baseline characteristics are presented in Table 1. The mean age of the cohort was 8.2 ± 4.1 years old (range [1.8–17.9]) and 66% of the cohort were male. Children with IgAVwoN were significantly younger (7.1 ± 4.0 years old) than patients in the HC group (9.9 ± 2.9 years old; *p* = 0.024). The incidence of hypertension was greater in the IgAVN group than in the IgAVwoN group (IgAVN—n = 4; IgAVwoN—n = 2; *p* = 0.013). Within the IgAVN group, 7 patients had histologically proven nephritis with a renal biopsy demonstrating IgA-positive staining on immunofluorescence. Of these, C3, IgG, and IgM positivity were present in six, two, and one patient biopsy samples, respectively. One patient had IgAVN previously (focal glomerulonephritis with <50% crescents—ISKDC grade IIIa). However, at study baseline, the UACR was less than 30 mg/mmol, hence for the purposes of this data they were categorised as IgAVwoN due to the inactive nephritis.

### 2.2. Urinary IgA Concentrations in Patients with IgAV and IgAVN

**Figure 1:**
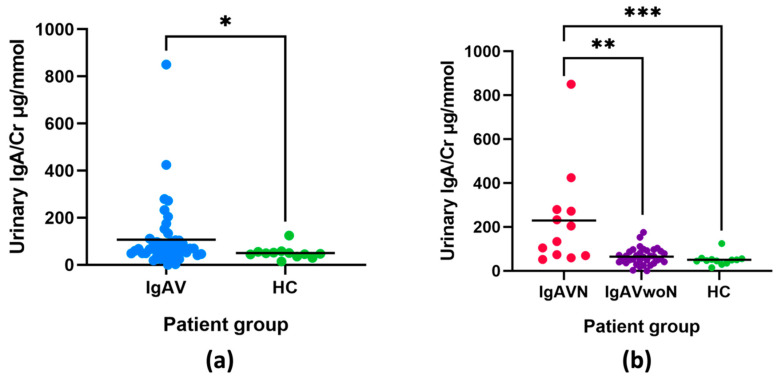
Urinary IgA/Cr concentrations between patients with IgAV compared to HC (**a**); between patients with IgAVN, IgAVwoN, and HC (**b**) with mean lines. Asterisks indicate a statistically significant difference between the groups (* *p* < 0.05, ** *p* < 0.01, *** *p* < 0.001). Results are normalised to urinary creatinine (Cr).

The urinary concentrations of IgA in patients with IgAV were compared to those in the HC group. The urinary IgA/Cr concentrations were statistically significantly increased by 2.1-fold in patients with IgAV (107.1 ± 136.3 μg/mmol) compared to HC (50.6 ± 26.3 μg/mmol; *p* = 0.027) (Figure 1a). Patients were then grouped according to the presence of nephritis to identify any difference in the urinary concentrations of IgA, results are presented in Figure 1b. The urinary IgA/Cr concentrations were statistically significantly increased by 3.5-fold in patients with IgAVN (229.8 ± 226.3 μg/mmol) compared to IgAVwoN (65.0 ± 37.8 μg/mmol]; *p* = 0.002) and by 4.5-fold in the IgAVN vs. HC comparison (50.6 ± 26.3 μg/mmol; *p* < 0.001). When the biopsy-proven IgAVN patients (n = 7) were stratified based on their histological findings, the numbers were too small for statistical comparisons, however, no evident trend was identified between the pathological findings and the urinary IgA/Cr concentrations, although the patient with more established sclerosis had a lower value (Table 2). 

### 2.3. Correlation of Urinary IgA with the Degree of Proteinuria

**Figure 2:**
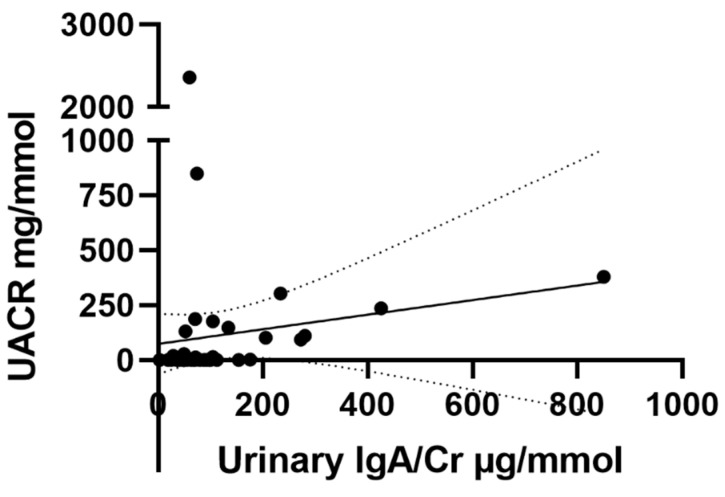
Correlation between urinary IgA/Cr and UACR in patients with IgAV with a linear regression best fit line (r = 0.124; *p* = 0.407) and 95% confidence bands. UACR: urinary albumin to creatinine ratio.

In the children with IgAV, the quantity of proteinuria defined using the urine albumin:creatinine ratio was compared to the urinary IgA concentration. As demonstrated in Figure 2, there was no statistically significant correlation observed between urinary IgA and the extent of proteinuria (r = 0.124; *p* = 0.407).

### 2.4. Serum IgA Concentrations in Patients with IgAV and IgAVN

**Figure 3:**
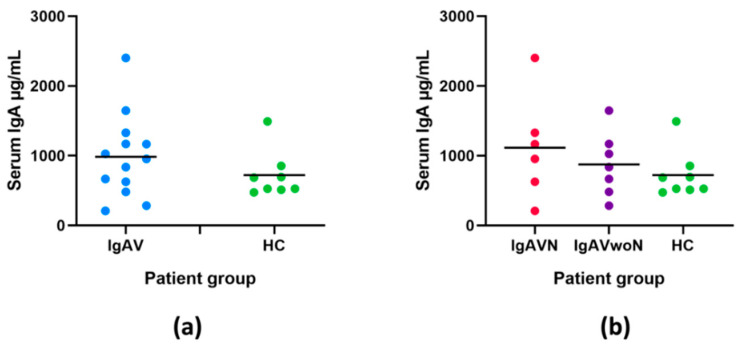
Serum IgA concentrations in patients with IgAV compared to HC (**a**) and in patients with IgAVN, IgAVwoN, and HC (**b**) with mean lines.

**Figure 4:**
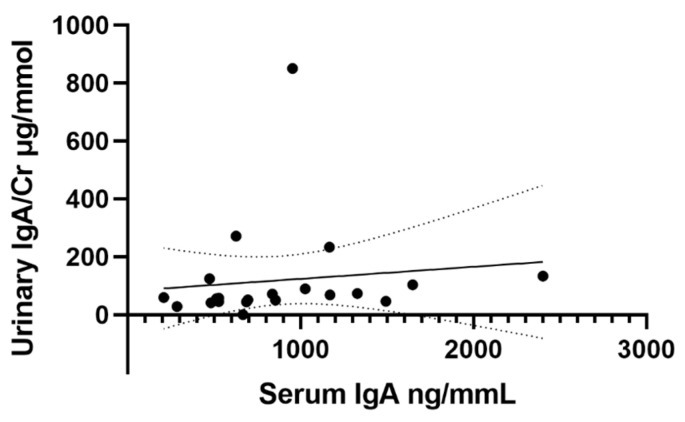
Correlation between urinary IgA/Cr and serum IgA concentrations in all patients with linear regression best-fit lines (r = 0.120; *p* = 0.603) and 95% confidence bands.

In a subgroup of 21 children for whom serum samples were available (IgAVN, n = 6; IgAVwoN, n = 7; HC, n = 8), the IgA serum concentrations were evaluated (Figure 3). There was no statistically significant difference between the serum IgA concentrations in all children with IgAV (983.8 ± 593.7 μg/mL) compared to HC (720.5 ± 337.3 μg/mL; *p* = 0.336) or between groups when patients were stratified according to the presence of nephritis (IgAVN—1113.0 ± 746.6 μg/mL; IgAVwoN—872.5 ± 457.4 μg/mL; HC—720.5 ± 337.3; *p* = 0.544). The urinary IgA/Cr concentrations did not correlate with the serum IgA concentrations (r = 0.120; *p* = 0.603) (Figure 4).

### 2.5. The Ability of Urinary IgA to Discriminate Patients with Nephritis

**Figure 5:**
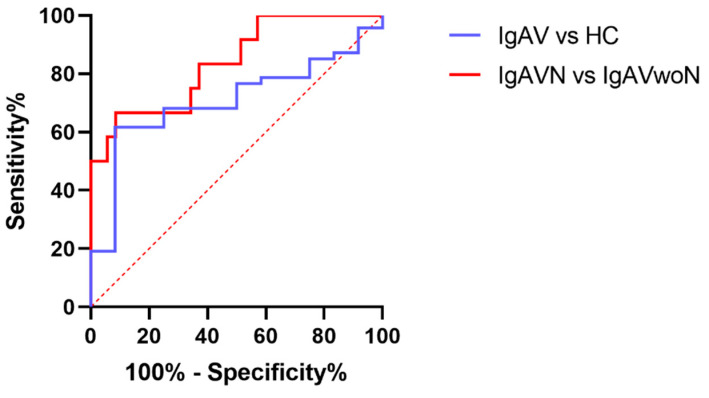
Receiver operating characteristic (ROC) curve analyses. ROC curves for the ability of urinary IgA to discriminate IgAV patients from HC, and IgAVN from IgAVwoN.

Receiver operating characteristic (ROC) curves were generated to assess the ability of urinary IgA to discriminate between all patients with IgAV compared to HC and between patients with IgAVN compared to IgAVwoN (Figure 5). Urinary IgA was acceptable at distinguishing patients with IgAV from HC (AUC 0.707, 95% CI [0.564–0.851], *p* = 0.028) and excellent at discriminating patients with and without nephritis (IgA—AUC 0.838, 95% CI [0.704–0.973], *p* < 0.001).

## 3. Discussion

This study aimed to measure the urinary concentrations of IgA in a cohort of children with IgAV in order to determine whether it can be used to distinguish between patients with and without nephritis. When patients were grouped according to the presence of nephritis, the urinary concentration of IgA was statistically significantly higher in the IgAVN group than in both the IgAVwoN and HC groups. Our results suggest that this finding doesn’t appear to be related to serum IgA concentrations or the extent of proteinuria. These results support previous work highlighting the potential utility of urinary IgA in determining patients with nephritis, which would need to be confirmed in larger longitudinal studies. The finding of elevated urinary IgA/Cr concentrations in patients with IgAVN is not that unexpected given that the pathophysiology is characterised by mesangial deposition of IgA1-containing immune complexes [7]. Pillebout and colleagues were the first to report increased urinary IgA concentrations in children with IgAVN, and they also found that the urinary IgA/Cr performed very well at identifying patients with nephritis (AUC 0.86; 95% CI [0.75–0.96]; *p* < 0.0001), with area under the curve values aligned with our findings. Interestingly, they reported a significant correlation of IgA/Cr with the concentration of proteinuria [8], which was not in keeping with our findings, but it is important to note that the correlation they reported was statistically significant (*p* < 0.006) but a relatively weak correlation (r = 0.47) [8,9]. In a prospective cohort of adults with IgAV, urinary IgA levels were also good predictors of poor kidney outcomes, with an optimum urinary IgA/Cr cut-off of 1.13 g/mmol (sensitivity of 76.2%, specificity of 80.0%) [10]. Other studies analysing urinary IgA have reported elevated concentrations in adult patients with IgA-related glomerulonephritis (IgAVN and IgA nephropathy) and found that IgA is predominantly excreted in a monomeric form [11,12,13]. Some adult studies report a positive correlation between the urine IgA concentration and total proteinuria [11,12]. However, determining the true relationship between any urine biomarker and total protein excretion is very challenging due to the lack of independence, as the definition of active nephritis often relies on the presence of proteinuria. Furthermore, Matousovic and colleagues added complexity to understanding the mechanisms of urinary IgA excretion by offering the theory that the aberrantly glycosylated hinge region may affect the net isoelectric charge of IgA, resulting in modified excretion, as the filtration of proteins through the glomerulus is thought to be charge-dependent [12].

Further development from our findings would be exploring whether the urinary IgA consists of the gd-IgA1 form and its immune complexes, as these have been proposed to be specific and prognostic markers of IgAVN. In previous reports, urinary IgA-sCD89 complexes were increased in children with IgAV compared to HC, whereas concentrations of IgA-IgG complexes were only elevated in the urine of children with nephritis [8]. This was also replicated in a Chinese study which described an incidence of 73% for IgAVN in hospitalised children with a urinary gd-IgA1/Cr ratio ≥ 105.74 U/mL during the six-month follow up [14]. Similar findings have been reported for IgAN, with some specificity of urinary gd-IgA1 to differentiate it from other kidney diseases [12,13,15].

With regards to the role of serum IgA, some studies have reported that serum IgA concentration is associated with nephritis in children with IgAV [8,16], but results are conflicting [11,17]. Our study found no significant difference in the serum IgA analyses, and a previous meta-analysis concluded that increased serum IgA levels were not associated with kidney involvement in IgAV [18]. These findings support the concept that urine is the preferred bio-fluid to help understand the evolution of nephritis in this condition [12,13]. 

This study has some limitations, which include its cross-sectional nature and heterogeneous cohort, which did not allow for a comparison over time such as that between the acute disease phase and remission. The time from sampling was also variable; however, post hoc analysis did not demonstrate any statistically significant differences in the urinary IgA concentrations and the time from diagnosis. This was also a single centre with relatively small cohort; hence, we were unable to truly assess the prognostic value of urinary IgA in terms of long-term outcomes. The definition of nephritis intentionally focused on the degree of proteinuria present due to its correlation with long-term kidney prognosis in the literature; however, we are aware that haematuria is included in the EULAR/PRINTO/PReS classification criteria. It is unknown whether the addition of haematuria would have strengthened the data findings. Similarly, the correlation between the histological findings and the urinary IgA concentrations is difficult to evaluate in this category of patients due to the very small number of biopsies performed. RAS inhibition or steroid treatment in a few of the patients may also represent a possible cofounder. Despite these limitations, this study, alongside existing evidence, opens up new areas for future work. Combining biomarkers such as urinary IgA with other novel markers or existing monitoring regimens that may be focused on urine dipstick findings may allow better information to identify children developing nephritis, and further scientific studies may utilise extracting IgA from urine to explore the IgA glycan profiles and better characterise this disease [15,19,20]. 

## 4. Materials & Methods

### 4.1. Patient Selection and Definitions

Children were recruited as part of the IgA Vasculitis Study, a single-centre observational study at Alder Hey Children’s NHS Foundation Trust, between August 28, 2019 and September 15, 2021. Children of any sex aged <18 years old at first presentation with a diagnosis of IgAV according to the EULAR/PRINTO/PReS 2008 criteria [21] were eligible to take part. Exclusion criteria were as follows: (i) diagnosis of IgAV uncertain; (ii) other concurrent inflammatory or renal condition; (iii) undergoing dialysis; (iv) urine infection, or; (v) no urine sample available. As this study was cross-sectional, a clean-catch, midstream spot urine sample could be obtained at any point during the follow-up period.

Patients were grouped according to the presence of renal involvement into either IgAVN (IgAV nephritis group) or IgAVwoN (IgAV without active nephritis group). IgAVN was defined based on the degree of proteinuria as a urinary albumin to creatinine ratio (UACR) of >30 mg/mmol at the time of sampling [21]. Any renal histology was graded according to the International Study of Kidney Disease in Children (ISKDC) classification for IgAVN [22]. Hypertension was defined as a systolic blood pressure above the 95th centile for the child’s age, sex, and height for <16 years old or >140 mmHg for children 16 years and older [23].

Healthy controls (HC) were recruited to provide age and sex-matched urine samples as per the IgA Vasculitis study protocol. They were children (aged < 18 years old) with no relevant past medical history (i.e., no history of autoimmune or renal disease) and not taking any regular medication; attending for day-case investigations or surgery.

### 4.2. Data Collection

Demographics and clinical data were collected at the time of the sample collection to provide baseline clinical characteristics. This included sex, age, blood pressure, height, serum creatinine (if available), UACR, a renal histology report, and any medication. A UACR of 0 mg/mmol was assumed for patients with a urine dipstick result negative for protein.

### 4.3. Sample Processing

Healthy control urine samples were tested for bacterial contamination using urine dipstick testing, and they were discarded if they demonstrated positivity for leukocytes, nitrites, blood, or > +1 for protein. Urine and serum samples were centrifuged twice at 300× *g* for 10 min and stored at −80 °C. Samples were thawed at room temperature on the day of the experiment and vortexed for 10 secs immediately before use.

### 4.4. Assays

The determination of serum IgA and urinary IgA was performed using commercially available enzyme-linked immunosorbent assay (ELISA) kits (Bio-Techne, Abingdon, UK) as per the manufacturer’s instructions. A 50,000-fold dilution was used for the serum samples, and a 10-fold dilution was used for the urine samples. First, 50 µL of samples or standards were loaded in duplicate into each well and left to incubate for 2 h at room temperature (RT). The plates were then washed, incubated with 50 µL of IgA detection antibody for one hour at RT, washed again, and loaded with 50 µL of streptavidin-peroxidase conjugate. After 30 min of incubation at RT, the plates were loaded with 50 µL of chromogen reagent and left to incubate for 6 min. Following the addition of the stop solution, the plates were read on a microplate reader at 450 nm with a 570 mm wavelength correction on the POLARstar Omega device (BMG LABTECH GmbH, Ortenberg, Germany).

### 4.5. Creatinine Quantification

Urinary concentrations of IgA were corrected for the urinary creatinine concentrations. Automated quantification of urinary creatinine was performed by the Biochemistry Department (Alder Hey Children’s NHS Foundation Trust, Liverpool, UK) using a previously described enzymatic method [24] using an Abbott Architect Ci8200 clinical chemistry analyser (Abbott, Chicago, IL, USA).

### 4.6. Ethical Approval

All procedures involving human subjects were conducted in accordance with NIHR Good Clinical Practice, HTA Codes of Practice, the Declaration of Helsinki, and comparable ethical standards. This study was part of the IgA Vasculitis Study which was approved by HRA and Health and Care Research Wales (HCRW) on June 21, 2019 (REC 17/NE/0390, protocol UoL001347, IRAS 236599). Written informed consent was obtained from parents and children prior to any study-related procedure.

### 4.7. Data Analysis

The MARS Data Analysis Software, version 3.32 for Windows (BMG LABTECH GmbH, Ortenberg, Germany), was used for the data analysis. The mean value and standard deviation (SD) of the duplicates were calculated and corrected for dilution. Values that were below the lowest concentration of the standard points were imputed as (standard lowest concentration)/√ 2 following previously published convention [25]. For each comparison, the fold-change of the mean IgA concentrations was calculated. Data are presented as mean ± SD unless stated otherwise.

### 4.8. Statistical Analysis

The Statistical Package for the Social Science (SPSS) version 27.0 software for Windows (IBM Corp, Armonk, NY, USA) and GraphPad Prism version 8.0 for Windows (GraphPad Software, San Diego, CA, USA) were used for the statistical analysis. The data distribution was assessed using the Shapiro-Wilk test. The student t-test or one-way ANOVA with Tukey’s post-hoc test used for normally distributed data whereas the significance of non-normally distributed data was evaluated with the Mann-Whitney U test or Kruskal-Wallis with Dunn-Bonferroni post-hoc test. The Pearson’s chi square test was applied to continuous variables. Linear correlation was assessed with the Pearson’s correlation coefficient to assess the correlation between the urinary IgA concentrations and the UACR in patients with IgAV and between the urinary and serum IgA concentrations in all patients. A coefficient of <0.30 was considered negligible, 0.30–0.50 low correlation, 0.50–0.70 moderate correlation, 0.70–0.90 high correlation, and 0.90–1.00 very high correlation [9]. Receiver operating characteristic (ROC) curves were generated to evaluate the ability of these urinary proteins to discriminate between patients with and without renal involvement. An area under the curve (AUC) of <0.7 was considered non-discriminant, 0.7–0.8 acceptable, 0.8–0.9 excellent, and >0.9 outstanding [26]. A *p*-value of < 0.05 was considered statistically significant.

## 5. Conclusions

Using a large cohort of children with IgAV, this study demonstrates that urinary IgA concentrations are increased in children with IgAVN. These findings may support the potential utility of urinary IgA as a tool to further evaluate its glycosylation status and/or identify those at risk of nephritis.

## Figures and Tables

**Table 1:** Patient characteristics at baseline.

	Overall	IgAVN	IgAVwoN	HC	*p*-Value
n (%)	59 (100)	12 (20)	35 (59)	12 (20)	-
Male ^a^	39 (66)	7 (58)	23 (66)	9 (75)	0.687
Age, years ^b^	8.2 ± 4.1	9.9 ± 4.4	7.1 ± 4.0 *	9.9 ± 2.9	0.008
Weeks from diagnosis to sampling ^b^	23.9 ± 47.5	43.2 ± 84.2	17.3 ± 24.0	-	0.188
Renal involvement					
Hypertension ^a^	6 (13)	4 (33) ^+^	2 (6)	-	0.013
Serum creatinine mg/Dl ^b,c^	45.9 ± 17.2	52.6 ± 14.9	38.5 ± 17.1	-	0.056
UACR mmol/mg ^b^	109.7 ± 365.4	422.8 ± 644.0 ^+++^	2.3 ± 6.2	-	<0.001
Urinary creatinine mmol/L ^b^	7.9 ± 6.2	6.4 ± 5.1	7.5 ± 5.6	10.6 ± 8.4	0.21
Biopsy proven nephritis ^a^	-	7 (58)	1 (2.8)	-	-
ISKDC Grade					
II ^a^	-	2 (17)	-	-	-
IIIa ^a^	-	-	1 (100)	-	-
IIIb ^a^	-	4 (33)	-	-	-
IV ^a^	-	1 (8)	-	-	-
Medications	8 (17)	6 (50)	2 (5.6)		
Corticosteroids ^a^	6 (13)	5 (42)	1 (3)	-	<0.001
ACE inhibitors ^a^	3 (6)	2 (17)	1 (3)	-	0.091
DMARDs ^a^	4 (9)	3 (25)	1 (3)	-	0.018

^a^ n (%); ^b^ mean ± SD. ^c^ Serum creatinine was available for 23 patients (IgAVN, n = 12 and IgAVwoN, n = 11). UACR: urinary albumin to creatinine ratio. ISKDC: International Study for Kidney Disease in Children Classification. DMARDs: disease modifying anti-rheumatic drugs. DMARDs used in this cohort: hydroxychloroquine, mycophenolate mofetil, and azathioprine. Significant *p*-values are highlighted in bold. * *p* < 0.05 compared to HC; ^+^ *p* < 0.05 compared to IgAVwoN; ^+++^ *p* <0.001 compared to IgAVwoN. Due to rounding, percentages may not add up to 100.

**Table 2:** Descriptive table of urinary IgA/Cr concentrations in biopsy-proven IgAVN patients classified as per the histological findings.

Pathological Findings (ISKDC Score)	n	Urinary IgA/Cr μg/mmol ^a^
Mesangial proliferation (II)	2	132.2 ± 102.9
Diffuse proliferation or sclerosis with <50 crescents (IIIb)	4	228.0 ± 157.3
Diffuse proliferation or sclerosis with 50–75% crescents (IV)	1	70.0 ± 0.0

ISKDC: International Study for Kidney Disease in Children Classification. ^a^ mean ± SD.

## Data Availability

The datasets analysed in this study are available from the corresponding author on reasonable request.
